# Effects of Neuromuscular Priming with Spinal Cord Transcutaneous Stimulation on Lower Limb Motor Performance in Humans: A Randomized Crossover Sham-Controlled Trial

**DOI:** 10.3390/jcm14124143

**Published:** 2025-06-11

**Authors:** Simone Zaccaron, Lara Mari, Mattia D’Alleva, Jacopo Stafuzza, Maria Parpinel, Stefano Lazzer, Enrico Rejc

**Affiliations:** 1 Department of Medicine, University of Udine, 33100 Udine, Italy; simone.zaccaron@uniud.it (S.Z.); mari.lara@spes.uniud.it (L.M.); mattia.dalleva@uniud.it (M.D.); jacopo.stafuzza@uniud.it (J.S.); maria.parpinel@uniud.it (M.P.); stefano.lazzer@uniud.it (S.L.); 2 School of Sport Sciences, University of Udine, 33100 Udine, Italy; 3 Department of Neurosciences, Biomedicine and Movement Sciences, University of Verona, 37129 Verona, Italy

**Keywords:** electromyography, maximal explosive power, maximal voluntary contraction, motor control, neuromodulation

## Abstract

**Background**: Lower limb motor output contributes to determining functional performance in many motor tasks. This study investigated the effects of non-invasive spinal cord transcutaneous stimulation (scTS) applied during an exercise-based priming protocol on lower limb muscle force and power generation. **Methods**: Twelve young, physically active male volunteers (age: 22.7 ± 2.1 years) participated in this randomized crossover, sham-controlled study. The maximal voluntary contraction and low-level torque steadiness of knee extensors, as well as the maximal explosive extension of lower limbs, were assessed before and after the priming protocol with scTS or sham stimulation over a total of four experimental sessions. Further, characteristics of evoked potentials to scTS related to spinal circuitry excitability were assessed in the supine position before and after the scTS priming protocol. The exercise component of the ~25 min priming protocol consisted of low-volume, low- and high-intensity lower limb motor tasks. **Results**: scTS priming protocol tended to increase or maintain maximum isometric torque during knee extension (4.7%) as well as peak force (0.2%) and rate of force development (6.0%) during explosive lower limb extensions, whereas sham priming protocol tended to decrease them (−4.3%, −3.3%, and −15.1%, respectively). This resulted in significant interactions (*p* = 0.001 to 0.018) and medium–large differences between scTS and sham protocols. These findings were associated with meaningful trends of some neurophysiological variables. Conversely, priming protocols did not affect low-level torque steadiness. **Conclusions**: scTS counteracted the unexpected fatigue induced by the exercise-based priming protocol, supporting lower limb performance during maximal efforts. Future studies are warranted to assess the implementation of scTS with optimized exercise-based priming protocols during training and rehabilitation programmes that include high-intensity neuromuscular efforts.

## 1. Introduction

Muscle strength and power contribute to determining the performance of a variety of human motor tasks, ranging from activities of daily life to athletic capabilities in many sports [1,2]. Further, muscle strength demonstrates high predictivity for overall morbidity and mortality [3,4], and lower limb power output is a significant predictor of functional performance in ageing [5]. Strength and power are defined and limited by muscle mechanics, morphological factors, and neural factors [6]. In particular, neural drive to the muscles, defined as the sum of spiking activities of motor neurons, plays a crucial role in determining strength and power output [6,7,8]. For example, force output is generally limited by the inability of the nervous system to maximally drive the muscle (i.e., activation deficit) [9,10,11]. Neural drive and voluntary activation are also altered by central fatigue [9], which leads to a reduced recruitment and firing frequency of spinal motoneurons [12].

Over the past decades, non-invasive neuromodulation techniques, such as Transcranial Magnetic Stimulation (TMS) and Transcranial Direct Current Stimulation (tDCS), have been explored as potential tools for enhancing neuromuscular performance [13], with the goal of modulating the sustained neural drive to motor neurons to result in increased muscle activation and motor output [14]. Only a limited number of these studies were performed in athletes and the outcomes remain inconclusive, with some studies showing improvements in maximal voluntary contraction [15,16] and power output during a vertical jump [17], while others did not find significant changes [18] or even noted a deterioration in performance [19]. Such discrepancy is conceivably related to differences in experimental protocol, muscle groups, participants’ characteristics and their tolerance to pain [20,21]. The positive effects of non-invasive brain stimulation on neuromuscular performance are generally linked to a depolarization of the resting membrane potential of neurons, which would mediate changes in neural excitability and lead to increased spontaneous firing rate, thereby enhancing the neural drive to the working muscle [21,22,23].

The electrical stimulation of the lumbosacral spinal cord is another neuromodulation approach stemming from spinal cord injury (SCI) research, and its potential to modulate neuromuscular function and performance in able-bodied individuals is still largely unknown. In the past decade, the application of invasive and non-invasive tonic spinal cord stimulation has been combined with activity-based training in individuals with SCI, promoting remarkable recovery of lower limb motor function even in those diagnosed with a chronic, clinically motor complete injury [24,25,26,27]. In this population, spinal cord stimulation modulates, and often increases, the excitability of spinal circuitry so that residual supraspinal inputs crossing the lesion, which are not functional under normal circumstances (i.e., chronic paralysis), demonstrate an augmented effect to generate and modulate different motor tasks [24,25,26].

In able-bodied healthy individuals, exploratory studies that have investigated the effects of non-invasive spinal cord stimulation on motor control and neuromuscular performance are rather scanty. For instance, the effect of a transcutaneous spinal direct current stimulation or sham stimulation applied in supine position was tested during series of subsequent countermovement jumps performed up to 3 h after the application of spinal stimulation in two separate sessions [28]. The time course of the kinetic outcomes collected in this pilot study throughout the 3 h protocol suggested opposite trends between spinal stimulation (i.e., positive trends) and sham stimulation (negative trends). The transcutaneous spinal direct current stimulation applied during a single session of backward locomotor training also appeared to improve learning and retention of such locomotor skills, compared to backward locomotor training with sham stimulation [29]. Spinal cord transcutaneous stimulation (scTS) with 20 Hz stimulation frequency applied at midline over the lumbosacral spinal cord also appeared to interact with postural control strategies, impairing steady standing postural control [30]. Overall, the outcomes of these studies suggest that non-invasive spinal cord stimulation can interact with the spinal circuitry controlling lower limbs in able-bodied subjects; however, the related mechanisms and effects on neuromuscular performance remain uncertain.

Here, our main goal was to assess whether spinal cord transcutaneous stimulation (scTS) can be a viable option to enhance maximal neuromuscular performance of lower limbs in young, physically active individuals. In order to achieve this effect, we opted to apply scTS during an exercise-based protocol (i.e., ~25 min of low-volume, low- and high-intensity lower limb motor tasks, [31]) so that spinal cord stimulation, afferent and supraspinal inputs to the spinal cord would be integrated to prime the nervous system. Our mechanistic hypothesis is that this approach could promote an increased excitability of the spinal circuitry, bringing the related neural network closer to activation threshold. This adaptation would amplify the effect of descending neural drive and possibly counteract the physiological activation deficit, which may range between 5% and 15% for key lower limb extensors (i.e., quadriceps femoris) in young healthy individuals [9,10,11,32].

Thus, we expected that the scTS priming protocol would promote enhanced lower limb force and power generation compared to the same exercise-based priming protocol performed with sham stimulation. Additionally, we assessed the effects of scTS and sham priming protocols on a submaximal, non-fatiguing torque steadiness task to test the secondary hypothesis that neuromuscular control of a finer motor task involving low-level and non-fatiguing efforts would not be differentially modulated by the two priming protocols.

## 2. Materials and Methods

### 2.1. Research Participants

Twelve healthy and physically active young male individuals, whose characteristics are reported in Table 1, were recruited at the School of Sport Sciences (University of Udine, Italy).

Inclusion criteria to participate in this study were: (i) age between 18 and 40 years; (ii) males; (iii) body mass index ranging from 18.5 to 29.9 kg/m^2^. Exclusion criteria were: (i) presence of surgically implanted neurostimulators and/or metal implants at the trunk and pelvis; (ii) acute and/or chronic clinical conditions; (iii) contraindications to perform physical exercise; (iv) having suffered from seizures; (v) psychiatric disorder or drug abuse. The research participants were non-professional athletes who practiced team sports, individual sports and/or resistance training activities (Table 1). The subjects had no history of neurological and orthopedic injuries. The experimental protocol was conducted in accordance with the Declaration of Helsinki and was approved by the Institutional Review Boards of the University of Udine (IRB# 197/2023) on 4 October 2023. Before the start of the study, subjects were carefully informed about its purpose and risks, and written informed consent was obtained from all of them.

### 2.2. Experimental Protocol

**Figure 1:**
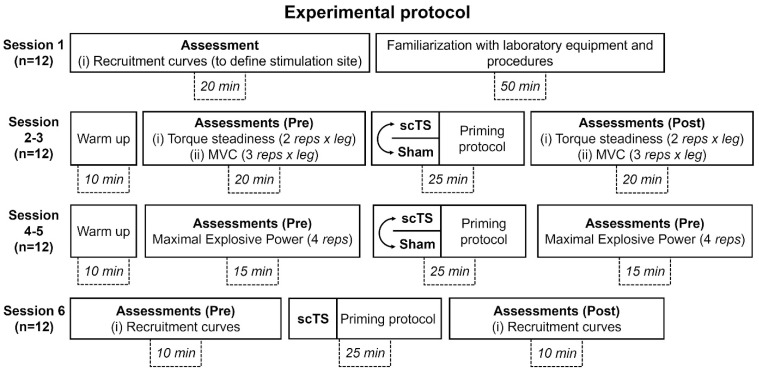
Experimental protocol. Overview of the six experimental sessions of the study. All subjects enrolled (n = 12) completed the experimental protocol. Testing of scTS or sham priming protocol in Sessions 2–3 and 4–5 was proposed in a randomized order. Approximate duration of the portions of each experimental session (participant’s preparation excluded) is reported in minutes. Reps: number of repetitions performed for the tested motor task. MVC: maximal voluntary contraction; scTS: spinal cord transcutaneous stimulation; Sham: sham stimulation.

The experimental protocol of this randomized crossover, sham-controlled study comprised six visits to the laboratory (Figure 1). Each session lasted approximately between 1 h (Session 6) and 1 h 30 min (Sessions 2–3) including participant’s preparation, and the time interval between consecutive sessions ranged between 2 and 5 days.

The first experimental session was devoted to (i) the assessment of anthropometric characteristics; (ii) determining the preferred stimulation site by assessing the relationship between scTS intensity and spinal cord-evoked potentials characteristics of lower limb muscles (i.e., recruitment curves); and (iii) the familiarization of participants with the laboratory equipment and procedures of the study, such as the application of scTS, isometric knee extensions on the dedicated ergometer, and explosive lower limb extensions on a sled ergometer. The second and third sessions were devoted to assessing the effects of the priming protocol with scTS or sham stimulation (in a randomized order) on maximal voluntary isometric contractions (MVC) and low-level isometric torque steadiness during knee extension. Similarly, during the fourth and fifth sessions, we examined how the priming protocol with scTS or sham stimulation (in a randomized order) affected the maximal explosive power of lower limbs. Finally, the sixth session explored the effects of the scTS priming protocol on the characteristics of the recruitment curves assessed with the participants relaxed in supine position.

### 2.3. Data Acquisition and Experimental Procedures

#### 2.3.1. Anthropometric Characteristics

Body mass was measured to the nearest 0.1 kg using a manual weighing scale (Seca 709, Hamburg, Germany) with the subject wearing only light underwear and no shoes. Stature was measured to the nearest 0.5 cm on a standardized wall-mounted height board. The dominant lower limb was considered as the limb used to kick a ball [33].

#### 2.3.2. Data Acquisition System

Electromyography (EMG) and kinetic signals, as well as the onset of each stimulation pulse, were collected using a dedicated acquisition system (Smart DX I, BTS Bioengineering, Milan, Italy) with a sampling rate of 1000 Hz, and the related software (Smart Motion Capture System Version: 1.10.0469, BTS Bioengineering, Milan, Italy).

#### 2.3.3. Surface EMG Recordings

Surface EMG was collected using a wireless EMG system (BTS FREEEMG1000, BTS Bioengineering, Milan, Italy; Input impedance: 100 MΩ; Common Mode Rejection Ratio: >110 dB @50–60 Hz; Sensitivity: 1 µV) and pre-gelled surface electrodes (BlueSensor N-00-S/25, Ambu, Penang, Malaysia) placed with inter-electrode distance equal to 20 mm. To ensure a good electrode–skin interface, prior to the application of the electrodes, the subject’s skin was shaved, rubbed with an abrasive paste, cleaned with an alcohol solution, and dry-cleaned with gauze. EMG electrodes were fixed at the beginning of the experimental session and were not removed until the end of the session. EMG was collected from the following lower limb muscles: vastus lateralis (VL), at two-thirds on the line from the anterior spina iliaca superior to the lateral side of the patella; rectus femoris (RF), midway between the anterior spina iliaca superior and the superior part of the patella; medial gastrocnemius (MG), on the most prominent bulge of the muscle; and tibialis anterior (TA), at one-third on the line between the tip of the fibula and the tip of the medial malleolus [34].

#### 2.3.4. Isometric Knee Extension: Torque Steadiness and MVC

A custom-built chair ergometer instrumented with a torque sensor for each of the two attachments was connected to the acquisition system to assess torque steadiness and MVC during knee extension. The subject was seated on the chair with his trunk secured in an upright position with a dedicated belt in order to prevent any movement. The knee angle of the left and right lower limb was set at 100 degrees by properly positioning the chair attachments, and a strap was tightened around each ankle to prevent movement. During the torque steadiness trials, real-time visual feedback depicting the real-time torque exertion and the torque target (20% MVC, based on data collected during the first experimental session) was provided on a monitor positioned in front of the subject. Participants were instructed to gradually achieve the torque target and maintain the torque signal on the torque target line with the least possible error for the subsequent 15 s. Two attempts for each lower limb were performed, with a 1.5 min rest in between attempts. The subjects were subsequently asked to perform three 4–5 s MVCs for each lower limb (alternating right and left limb) as fast and forcefully as possible, with a 2 min rest between attempts.

#### 2.3.5. Maximal Explosive Power

The Explosive Ergometer (EXER), described in detail by previous work from our group [35] was used to assess the maximal explosive power of the lower limbs. Briefly, the EXER consists of a metal frame supporting one rail, which was inclined by 20 degrees. A seat, fixed on a carriage, was free to move on the rail, with its velocity along the direction of motion being continuously recorded by a wire tachometer (LIKA SGI, Vicenza, Italy). The total moving mass of the EXER (seat and carriage together) was equal to 31.6 kg. The subject was seated on the carriage seat, secured by a safety belt tightened around the shoulders and abdomen. Two mechanical blocks were used to set the distance between the seat and the force platforms (LAUMAS PA 300, Parma, Italy), so that the knee angle at rest was 100 degrees. The blocks also prevented any countermovement during the pushing phase. Participants placed their feet sole against the force platforms in a flat standardized position, and were instructed to perform a total of four maximal bilateral lower limb extensions as fast and forcefully as possible. When the subject performed an explosive effort, he and the seat moved backwards, and the force, velocity and EMG signals were collected by the acquisition system. After each push, the subjects rested for 2 min with their feet placed on a dedicated support.

#### 2.3.6. Spinal Cord Transcutaneous Stimulation

A constant current stimulator (DS7A, Digitimer, Hertfordshire, UK; maximal voltage: 400 V) was controlled by a trigger box (GeMS TRIGGER BOX, EMS, Bologna, Italy) and related software (Direct USB for TRIGGER BOX, Version 1.00, EMS, Bologna, Italy) to deliver the selected scTS protocols. Two 100 × 50 mm self-adhesive electrodes (20021, Axion GmbH, Leonberg, Germany) were placed symmetrically on the skin over the iliac crests as anodes [36]. Also, the cathode electrode was placed on the skin between either the T11-T12 or T12-L1 spinous processes, in the midline over the vertebral column, using a self-adhesive, circular electrode (diameter: 25 mm) (E-CM25, TensCare, Surrey, United Kingdom). An operator with approximately 10 years of experience identified the spaces between the spinous processes of the thoracic (T)11 and T12, and T12 and lumbar (L)1 vertebrae by palpation, which were marked with the participant standing in an upright position.

#### 2.3.7. Recruitment Curves by Spinal Cord Transcutaneous Stimulation

After the stimulating electrodes were secured onto the skin, the participant was placed in a supine position on a standard bedded table and remained relaxed during the assessment. Spinal cord transcutaneous stimulation was delivered as single, 1 ms monophasic square-wave pulses every 4 s. Stimulation intensity started at 5 mA and, using 5 mA increments, it was increased up to 100 mA, or the maximum intensity that did not result in discomfort for the participant. Five stimuli were delivered at each intensity. All subjects of this study achieved 100 mA as maximum stimulation intensity for all recruitment curve assessments.

During experimental session 1, the T11-T12 and T12-L1 stimulation sites were tested in a randomized order with a 4 min pause in between assessments. Recruitment curves were assessed to define the preferred stimulation site to be used for all subsequent experimental sessions (see Section 2.4.1 Data Analysis and Computation of Experimental Variables—Recruitment Curves for details). In order to maximize the reproducibility of cathode electrode placement across experimental sessions, the electrode location was marked on the skin with a permanent ink pen, and pictures of the electrode were also taken.

During experimental session 6, the same spinal stimulation procedure (using only the selected stimulation site) was performed at baseline and four minutes after the completion of the priming protocol with scTS to explore its effects on neurophysiological variables related to the excitability of the spinal circuitry (Figure 1).

#### 2.3.8. Neuromuscular Priming with scTS or Sham Stimulation

In the present study, an exercise-based intervention with scTS or sham stimulation, which lasted approximately 25 min, was implemented to prime the neuromuscular system with the goal of modulating lower limb neuromuscular performance. With the participant in standing position, stimulating electrodes were placed onto the skin over the iliac crests (anodes) and the selected intervertebral space (cathode; see Section 2.3.6 Spinal Cord Transcutaneous Stimulation), and were secured by elastic bandages wrapped around the participant’s trunk. A small foam piece was placed between the cathode electrode and the bandage to maintain pressure on the electrode.

Tonic, monophasic scTS was delivered at 28 Hz with 1000 µs pulse width using the constant current stimulator system described above. During scTS, stimulation intensity was gradually increased to achieve the highest intensity that was well tolerated by the participant in terms of comfort and ability to perform the requested motor tasks without any restriction. On average, stimulation intensity applied during the neuromuscular priming protocols with scTS was 25.4 ± 4.4 mA. scTS was well tolerated by all participants but one, who frequently reported an itchy sensation in the abdomen area around the electrodes; nevertheless, he completed the entire study protocol. No visible lower limb muscle contraction was elicited by scTS. On the other hand, during sham stimulation, intensity was gradually increased for only one minute, followed by a 10 s decrease and the subsequent stimulator turning off; thus, no stimulation was delivered for the remaining ~24 min of the sham priming protocol [37]. While we did not assess credibility and expectance of sham stimulation, three participants self-reported perception of spinal stimulation at the end of a sham session. In particular, “whole-body pinching sensation” and “perception of stimulation when increasing attentiveness” were reported.

The priming protocol included an initial quiet standing period with increasing scTS intensity, followed by the practice of stepping in place, unilateral balance control and unilateral quarter squats. Generally, 3 min of low-intensity exercise were interleaved with 30 s of quiet standing to complete a set; a total of three sets were performed. Finally, high-intensity attempts of the motor task tested during the session (i.e., n = 3 isometric knee extensions per limb in an alternated fashion, or n = 4 explosive lower limb extensions) were performed with a 2 min rest in between attempts. During experimental session 6 (i.e., recruitment curves before and after neuromuscular priming protocol), isometric knee extensions were included in the priming protocol.

### 2.4. Data Analysis and Computation of Experimental Variables

Data were processed using the software LabChart Reader ver. 8.1.3 (ADInstruments, Inc., Dunedin, New Zealand). EMG signals were band pass-filtered at 10–499 Hz. Torque signals collected during isometric assessments were lowpass filtered at 10 Hz. Force and velocity signals collected from the EXER during lower limb extensions were lowpass filtered at 25 Hz.

The data processing implemented to compute the experimental variables of interest for each assessment is detailed here below. Only the best attempts (i.e., lowest coefficient of variation during torque steadiness; highest torque during MVC; highest maximal explosive power during explosive extensions) within each experimental condition (scTS or sham stimulation) and part of the session (before or after the priming protocol) were taken into consideration for further analysis.

#### 2.4.1. Recruitment Curves

Peak-to-peak amplitude of the evoked potentials to spinal stimulation was quantified. The average EMG peak-to-peak amplitude for each stimulation intensity (i.e., five stimuli were delivered at each stimulation intensity) was calculated. Muscle activation threshold coincided with the lowest stimulation intensity that induced reproducible evoked potentials with amplitude higher than the mean baseline EMG plus three times its standard deviation [38]. The largest EMG peak-to-peak amplitude was also considered for analysis.

For each participant, the stimulation site (i.e., T11-T12 or T12-L1 spinous processes) favouring the overall lower activation threshold and larger EMG peak-to-peak amplitude for the right and left VL and MG was selected and used for all subsequent sessions. For all participants, T12-L1 was selected as the stimulation site. When assessing the effects of the priming protocol on the characteristics of the evoked potentials to spinal stimulation (experimental session 6), the right and left VL were considered for analysis, and EMG peak-to-peak amplitude was expressed as percentage of the largest amplitude generated before (Pre) or after (Post) the priming protocol within each muscle and research participant. Data deriving from the left and right VL were averaged and then considered for the Post- vs. Pre-priming protocol analysis.

#### 2.4.2. Torque Steadiness

The variability of torque generated during low-level (20%MVC) isometric knee extension was assessed by calculating its coefficient of variation (standard deviation/mean) over a 10 s sliding window that considered the ~20 s attempt. The time window returning the lowest coefficient of variation was also used to assess the EMG amplitude (by root mean square) as well as median power frequency for the knee extensors considered in this study (VL and RF). EMG amplitude was expressed as percent of the MVC obtained within the same experimental session. EMG amplitude and median power frequency of VL and RF were averaged to assess the overall behaviour of knee extensors (KE) [35,39]. Data deriving from the right and left lower limb were finally averaged to assess the overall effects of the priming protocol with scTS or sham stimulation.

#### 2.4.3. MVC

The maximum torque generated during isometric knee extension was defined by a 1 s sliding window. EMG amplitude and median power frequency of VL and RF were also calculated within the 1 s window corresponding to maximum torque output. The rate of torque development was characterized by its peak slope detected during torque rising. EMG amplitude was expressed as a percent of the MVC obtained for each muscle and research participant within the same experimental session. EMG amplitude and median power frequency of VL and RF were averaged to assess the overall behaviour of KE. Data deriving from the right and left lower limb were finally averaged to assess the overall effects of the priming protocol with scTS or sham stimulation.

#### 2.4.4. Maximal Explosive Power

Mechanical power developed during the maximal bilateral explosive extensions on the EXER was obtained from the instantaneous product of the bilateral forces multiplied by the backward velocity. Peak force, rate of force development, velocity, and power were considered for analysis. EMG amplitude of VL, RF, MG, and TA of the dominant lower limb was characterized by root mean square during the push phase (i.e., throughout the period of force development), and expressed as percent of isometric MVC performed at the beginning of the experimental session. EMG amplitude of VL and RF were averaged to assess the overall behaviour of KE.

#### 2.4.5. Statistical Analysis

Statistical analysis was performed using JASP 0.19 (Amsterdam, The Netherlands). A *p* value less than 0.05 was considered statistically significant. Results were expressed in figures and text as mean and standard error. The Shapiro–Wilk test was used to verify the normality of distributions. Two-way within-subjects ANOVA was implemented, with “Time” (i.e., Pre vs. Post neuromuscular priming protocol) and “Treatment” (i.e., priming protocol with scTS or sham stimulation) as repeated measures factors. Assumption of sphericity was met, as factors with only two levels of repeated measures were included in the analysis. When significant differences were found, a Bonferroni post hoc test was used to determine the exact location of the differences. Also, a paired *t*-test or Wilcoxon test, depending on data distributions, was used to compare the Post vs. Pre percent difference (Post-Pre-Δ%) promoted by the scTS or sham priming protocol. Finally, effect sizes (ES) comparing Post-Pre-Δ% values obtained with the scTS or Sham priming protocol were calculated. ES values lower than 0.20 were considered negligible, between 0.20 and 0.49 small, between 0.50 and 0.79 medium, and equal or greater than 0.80 large [40]. The sample size for this study was estimated a priori using G*power (version 3.1.9.7, Henry University of Düsseldorf, Düsseldorf, Germany) [41]. In particular, a sample size of 12 was sufficient to detect an ES of 0.9 achieving over 80% of power for a 2-sided test with matched pairs (i.e., Post-Pre-Δ% obtained with the scTS or sham priming) with a significance level of 0.05.

## 3. Results

**Figure 2:**
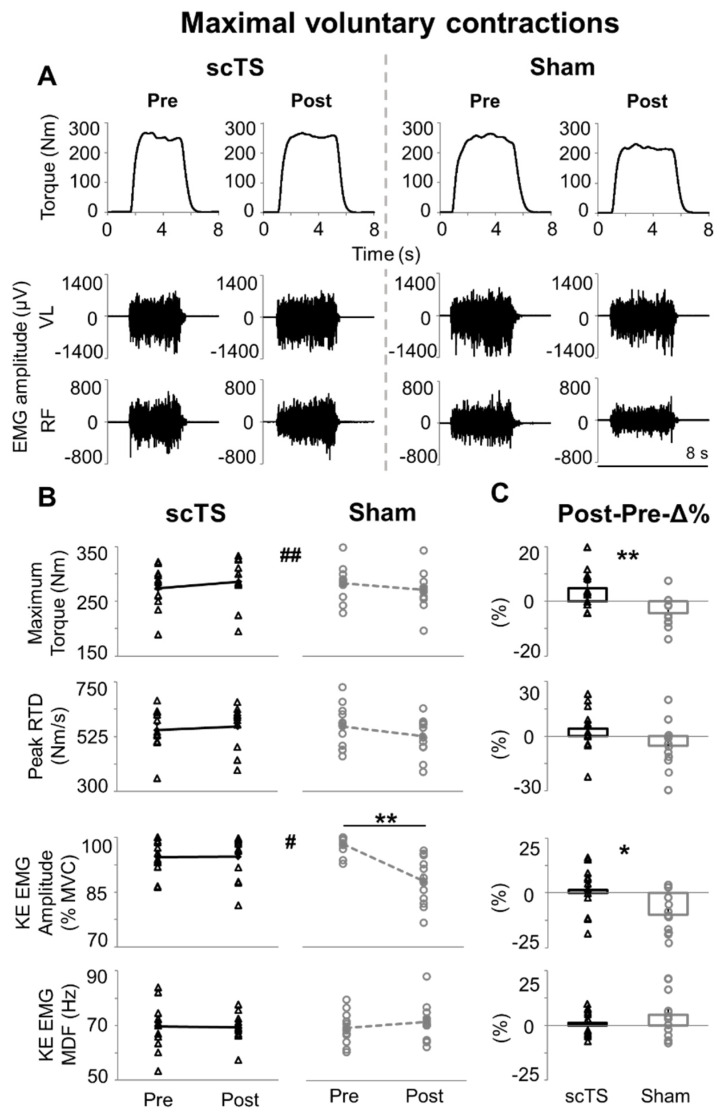
Maximal voluntary contractions. (**A**) Time course of torque output and EMG activity of vastus lateralis (VL) and rectus femoris (RF) during representative maximal voluntary isometric knee extensions generate before (Pre) and after (Post) priming protocol with spinal cord transcutaneous stimulation (scTS) or sham stimulation (Sham). (**B**) Maximum torque output, peak rate of torque development (RTD), EMG amplitude (quantified by root mean square) and median power frequency (MDF) of knee extensors (KE, average value between VL and RF). Significant Time × Treatment interaction by two-way within-subjects ANOVA: # *p* < 0.05; ## *p* < 0.01. ** significant difference by Bonferroni post hoc test, *p* < 0.01. (**C**) Post vs. Pre percent difference (Post-Pre-Δ%) assessed within the scTS or sham priming session were statistically compared by paired *t*-test or Wilcoxon test: * *p* < 0.05; ** *p* < 0.01. Results in (**B**,**C**) are reported as individual data points (empty black triangles: scTS session; empty grey circles: sham session) as well as mean and standard error. Note that individual data points are superimposed on error bars.

During the two experimental sessions devoted to MVC assessment, maximum isometric torque generated by knee extensors and EMG activity of VL and RF were collected Pre and Post priming protocol with scTS or sham stimulation (Figure 2A). No significant Treatment effect was observed for MVC-related variables, with *p* values ranging between 0.261 and 0.643 (KE EMG amplitude and median power frequency, respectively; Figure 2B). Conversely, significant Time × Treatment interaction (*p* = 0.001) was found for the maximum torque generated, with post hoc analysis revealing a trend (*p* = 0.070) of lower maximum torque at Post-Sham compared to Pre-Sham. Accordingly, Post-Pre-Δ% of maximum torque was significantly different (*p* = 0.002, ES: 1.19; Figure 2C) between scTS and sham priming protocol, with a positive trend (4.67 ± 0.07%) observed after scTS priming protocol while negative values (−4.30 ± 0.06%) were found in the Sham session. A similar trend was observed for RTD (Time × Treatment interaction *p* = 0.062), with Post-Pre-Δ% RTD values tending to be higher (*p* = 0.070, ES: 0.58) in the scTS priming sessions compared to the sham session.

These torque-related outcomes were supported overall by the EMG amplitude analysis performed on knee extensors. In particular, a significant Time × Treatment interaction (*p* = 0.035) was found for KE EMG amplitude, with post hoc analysis indicating lower values at Post-Sham (88.1 ± 1.9%MVC) compared to Pre-Sham (98.1 ± 0.7%MVC, *p* = 0.008; Figure 2B), whereas similar KE EMG amplitude values (*p* = 1.000) were found before and after the scTS priming protocol. Along this line, Post-Pre-Δ% values of KE EMG amplitude were higher in the scTS priming session compared to the sham session (*p* = 0.031, ES: 0.71; Figure 2C), with a mean difference of 11.1% between the two sessions. Conversely, KE EMG median power frequency was not affected by the neuromuscular priming protocols herein investigated (Time × Treatment interaction *p* = 0.282; Post-Pre-Δ% *p* = 0.286, ES: 0.32; Figure 2B,C).

**Figure 3:**
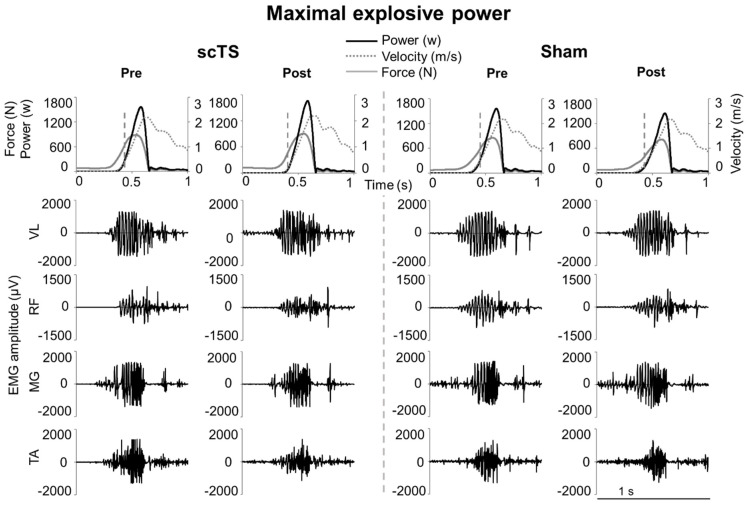
Maximal explosive power. Time course of force, velocity, power, and EMG of vastus lateralis (VL), rectus femoris (RF), medial gastrocnemius (MG), and tibialis anterior (TA) during representative bilateral explosive extensions of the lower limbs performed on the sled ergometer EXER, which was inclined by 20 degrees. Vertical, grey dashed lines in the top row indicate the time point corresponding to the peak rate of force development. These efforts were generated before (Pre) and after (Post) priming protocol with spinal cord transcutaneous stimulation (scTS) or sham stimulation (Sham).

A representative time course of the mechanical variables (force, velocity, power) and EMG activity of lower limb muscles during explosive efforts performed on the EXER, before and after the two neuromuscular priming protocols, is shown in Figure 3. 

**Figure 4:**
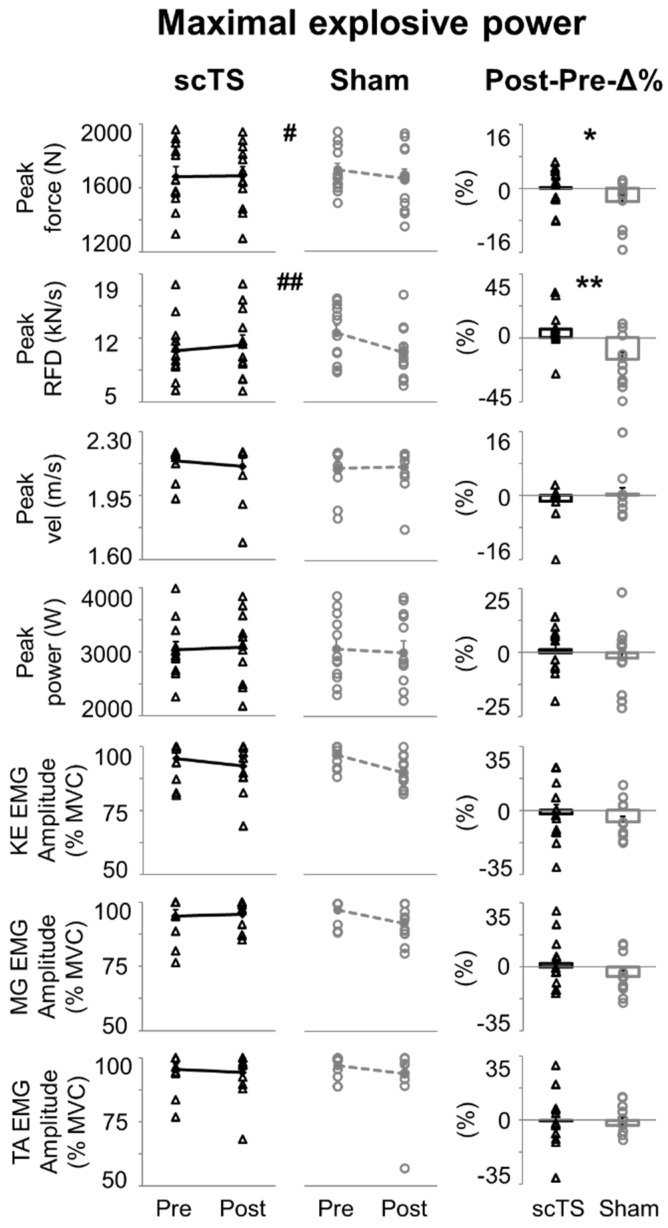
Maximal explosive power of lower limbs. Peak force, rate of force development (RFD), velocity, and power, as well as EMG amplitude (quantified by root mean square) of knee extensors (KE, average value between vastus lateralis and rectus femoris), medial gastrocnemius (MG) and tibialis anterior are reported as individual data points (empty black triangles: scTS session; empty grey circles: Sham session) or mean and standard error. Note that individual data points are superimposed on error bars. scTS: spinal cord transcutaneous stimulation; Sham: sham stimulation; Pre: before neuromuscular priming protocol; Post: after neuromuscular priming protocol; Post-Pre-Δ%: Post vs. Pre percent difference within each session. Significant Time × Treatment interaction by two-way within-subjects ANOVA: # *p* < 0.05; ## *p* < 0.01. Post-Pre-Δ% calculated within the scTS or sham priming session were statistically compared by paired *t*-test or Wilcoxon test: * *p* < 0.05; ** *p* < 0.01.

No significant Treatment or Time effect was observed for maximal explosive power-related variables, with *p* values ranging between 0.093 (KE EMG amplitude, Time effect) and 0.889 (MG EMG amplitude, Treatment effect). Conversely, a significant Time × Treatment interaction (*p* = 0.018) was found for the peak force, and the related Post-Pre-Δ% obtained in the scTS session was significantly higher than that assessed in the sham session (*p* = 0.019, ES: 0.79) (Figure 4). A similar finding was noted for peak RFD, which showed a significant Time × Treatment interaction (*p* = 0.003), and also a trend (*p* = 0.114) of lower values Post-Sham compared to Pre-Sham. Furthermore, for peak RFD, a large and significant difference between Post-Pre-Δ% obtained in the scTS and sham session was found (*p* = 0.002, ES: 1.13), with higher values in the former and a mean difference of 21.1% between the two sessions (Figure 4). No differences were observed for the other mechanical variables (peak velocity and power) considered in this assessment (Time × Treatment interaction *p* = 0.393 and 0.432, respectively; Figure 4). Similarly, no significant differences and only negligible or small effects were noted when assessing how EMG amplitude was affected by scTS or sham priming protocols (Figure 4).

**Figure 5:**
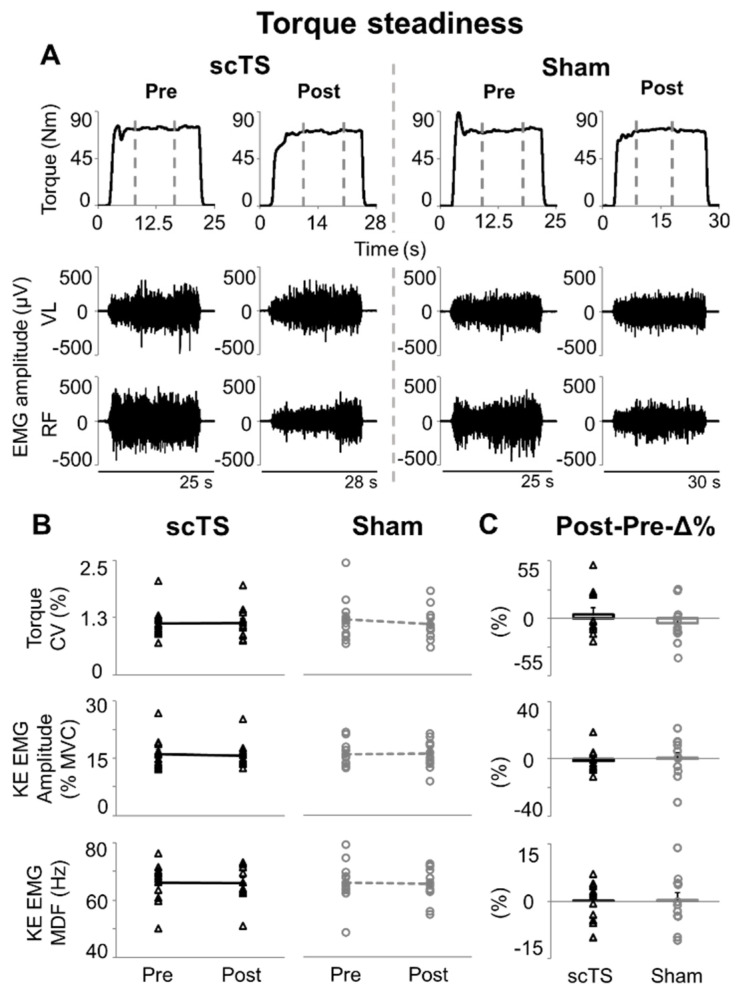
Torque steadiness. (**A**) Representative time course of torque output and EMG activity of vastus lateralis (VL) and rectus femoris (RF) during a 15-s isometric knee extension aiming at maintaining a 20% MVC torque output. Vertical grey dashed lines indicate the 10 s time windows with the lowest torque coefficient of variation that were considered for analysis. Attempts were performed before (Pre) and after (Post) priming protocol with spinal cord transcutaneous stimulation (scTS) or sham stimulation (Sham). (**B**) Torque coefficient of variation (CV), EMG amplitude (quantified by root mean square) and median power frequency (MDF) of knee extensors (KE, average value between VL and RF). (**C**) Post vs. Pre percent difference (Post-Pre-Δ%) assessed within the scTS or Sham priming session. Results in (**B**,**C**) are reported as individual data points (empty black triangles: scTS session; empty grey circles: Sham session) as well as mean and standard error. Note that individual data points are superimposed on error bars. No statistically significant difference found.

Effects of the neuromuscular priming protocols considered in this study on torque steadiness and EMG characteristics of knee extensors during low-level (20%MVC) efforts (Figure 5A) were overall negligible.

No significant Treatment or Time effect was observed for variables related to torque steadiness, with *p* values ranging between 0.176 (torque coefficient of variation, Time effect) and 0.824 (KE EMG amplitude, Treatment effect). Also, we found no significant Time × Treatment interactions nor meaningful trends for torque coefficient of variation (*p* = 0.275), EMG amplitude of KE (*p* = 0.393) and EMG median power frequency of KE (*p* = 0.857) (Figure 5B). Additionally, no differences between Post-Pre-Δ% outcomes assessed in the scTS or sham session were noted (torque coefficient of variation *p* = 0.402, ES: 0.25; KE EMG amplitude *p* = 0.630, ES: 0.14; KE EMG MDF *p* = 0.979, ES: 0.01; Figure 5C).

**Figure 6:**
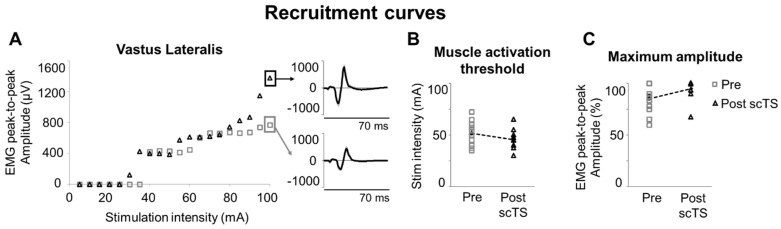
Recruitment curves. (**A**) Representative relationship between spinal cord transcutaneous stimulation (scTS) intensity and EMG peak-to-peak amplitude of vastus lateralis evoked potentials assessed before (Pre, grey empty squares) and after (Post, black empty triangles) scTS priming protocol. Each data point is the average peak-to-peak amplitude elicited by five stimuli. Exemplary evoked potentials to spinal stimulation (five individual responses are overlayed in grey; average response in black) corresponding to the maximum EMG peak-to-peak amplitude are also shown. (**B**) Vastus lateralis muscle activation threshold and (**C**) maximum EMG peak-to-peak amplitude assessed Pre and Post scTS priming protocol are reported as individual data points as well as mean and standard error. Note that individual data points are superimposed on error bars. No statistically significant difference found.

Finally, characteristics of the recruitment curves explored in supine position before and after the scTS priming protocol (which included high-intensity isometric knee extensions), revealed some trends that are worth being mentioned. In particular, VL muscle activation threshold tended to be lower after scTS priming protocol (*p* = 0.081, ES: 0.56; Figure 6A,B), with average stimulation intensity corresponding to activation threshold that decreased from 51.6 ± 3.2 mA to 45.5 ± 2.5 mA. Also, maximum VL EMG peak-to-peak amplitude tended to increase after scTS priming protocol (*p* = 0.071, ES: 0.53; Figure 6A,C), detecting average peak-to-peak amplitude of 84.6 ± 3.9% and 95.4 ± 2.7% at Pre and Post priming protocol, respectively.

## 4. Discussion

The neuromuscular priming protocol investigated in this study differentially affected key neuromuscular variables during maximal efforts of the lower limbs depending on whether scTS or sham stimulation was applied. Generally, scTS priming protocol tended to increase or maintain mechanical outputs such as the maximum isometric torque during knee extension or peak force and RFD during explosive lower limb extensions, whereas sham priming protocol tended to decrease them. These findings, resulting in significant, medium-large differences between priming protocols, were associated with meaningful trends of some of the neurophysiological variables investigated, such as the EMG amplitude of knee extensors during maximal voluntary contractions or recruitment curve outcomes related to the excitability of the spinal circuitry. Conversely, the two priming protocols had no effect on the submaximal motor task focused on steadiness of muscle torque generation.

### 4.1. Maximal Neuromuscular Performance of Lower Limbs

The descending neural drive is critical in determining the maximum torque generation by recruiting a larger amount of motor units of agonist muscles at their higher firing rate and optimal synchronization [42,43,44]. In the present study, neither neuromuscular priming protocol significantly affected maximum mechanical output within a given experimental session (i.e., Post vs. Pre priming protocol, Figure 2B). However, significant and meaningful interactions between the two priming protocols and their Post vs. Pre effects were noted, with trends of improved or maintained performance after scTS priming as compared to impaired performance after sham priming protocol (Figure 2B,C). Negative Post vs. Pre trends in the sham session can be interpreted as sign of neuromuscular fatigue. In this study, we did not implement neurophysiological assessments to directly examine and quantify central and peripheral components of fatigue. However, when considering the low volume of motor tasks requiring meaningful effort that were included in the priming protocol, the amount of rest between these efforts and their unilateral nature (thus doubling the rest time per leg), it is conceivable that central components primarily contributed to such negative trends. The significant decrease in KE EMG amplitude after the sham priming protocol (Figure 2B) further supports this perspective. Briefly, central fatigue is related to a reduced drive from the motor cortex to the muscles [45], leading to decreased EMG amplitude and maximum force generation [42,46], and can be responsible for approximately 20–25% of the loss of force associated with fatigue [9,47]. Afferent inputs deriving from muscle spindles, the Golgi tendon organ, as well as group III and IV fibres can also contribute to the reduction in spinal and corticospinal excitability [48].

The negative trends of neuromuscular performance induced by the exercise-based protocol implemented in this study (Figure 2 and Figure 4), conceivably related to central fatigue, were not hypothesized. Briefly, the exercise-based part of the priming protocol implemented in this study was characterized by low-volume and included general low-intensity tasks (e.g., unilateral half squat and balance control) as well as task-specific, high-intensity neuromuscular components (isometric knee extension or explosive lower limbs extension), which were aimed at positively affecting neural, muscular, and metabolic factors [31,49,50]. Muscle temperature modulation and increased muscle fibre sensitivity to calcium ions are a few of the potential mechanisms underlying exercise-based priming protocols. However, previous findings also indicate that performance responses to exercise-based neuromuscular priming are highly variable among individuals [31]. Additionally, individuals’ performance level may influence neuromuscular potentiation and its time course, as stronger individuals may elicit greater and earlier potentiation following neuromuscular priming [51].

Conversely, trends of enhanced or maintained neuromuscular performance were noted after scTS priming protocol (Figure 2B,C). scTS may have promoted an increased excitability of the spinal circuitry involved in control and activation of leg extensors, bringing the related neural network closer to activation threshold, thus amplifying the effect of the descending neural drive, which was conceivably impacted by fatigue, during MVC [52,53,54]. This mechanistic hypothesis originates from spinal cord injury studies showing that tonic, sub-motor threshold spinal stimulation can re-enable volitional leg movement in individuals with chronic clinically complete paralysis [55,56]. Also, it is worth noting that the two priming protocols promoted differential trends in KE EMG amplitude but similar trends in KE median power frequency (Figure 2B). This might suggest that priming protocols differentially affected the amount of motor units recruited during MVC without specificity in terms of their morpho-functional properties related to conduction velocity [57]. However, future dedicated investigation involving, for example, matrix EMG and decomposition algorithms is needed to address this mechanistic hypothesis.

Some mechanical outcomes assessed during explosive lower limb extensions presented similar trends to those found during MVC. In particular, the priming protocol differentially affected peak force and RFD depending on whether scTS or sham stimulation was applied (Figure 4), with negative Post vs. Pre trends found in the sham session and positive or no trends showed in the scTS priming session. Conversely, no significant interactions were found for peak velocity and power nor for EMG amplitude of the lower limb muscles considered for analysis. Isometric knee extension can be considered a rather simple motor task to control as it is focused on a single joint, it lacks meaningful joint movement, and its duration is relatively long (i.e., few seconds). Conversely, explosive lower limb extension performed on the EXER entails a relatively more complex motor control because it is a multi-articular, dynamic maximal effort that requires intra- and inter-muscular coordination over a very short push phase (~300 ms), which is generated against a fraction (approximately 48% in the present study) of body weight. Also, peak RFD and, to a lesser extent, peak force occur earlier in the push phase (i.e., corresponding to a slower instantaneous backward velocity) with respect to peak power and velocity (see Figure 3). From these observations, we may speculate that the spinal cord excitability modulation conceivably brought about by scTS favours motor outputs related to isometric efforts and to the earlier phase of a dynamic effort. Conversely, outputs related to the later and faster phase of a dynamic effort were not supported. The investigation of RFD at different time windows in relation to force generation onset, rather than the peak RFD considered in this study, would have provided additional details on this topic. For example, RFD assessed in the early phase (0–50 ms) of muscle contraction is of particular interest because it is primarily influenced by neural mechanisms [58,59]. However, a limit of the present study is that force (and torque during MVC) baseline signals were not sufficiently stable to implement a reliable detection of force generation onset to aim at such fine analysis.

### 4.2. Low-Level Torque Steadiness

Sub-maximal isometric contractions are key features of motor control, and the related steadiness of torque generation reflects the variance in descending neural drive and cumulative motor unit activity [60]. In the present study, neither priming protocol affected the torque steadiness during low-level contractions (Figure 5). EMG amplitude and median power frequency of KE were also not modulated by the priming protocols (Figure 5). These surface EMG variables provide rather limited insights into the specific control of muscle activation. However, their lack of modulation within our study is in line with previous research detailing the relationship between force fluctuations during torque steadiness and some characteristics of the motor units recruited, such as their number, their upper limit of recruitment and their contractile properties [60]. Also, it is important to consider that the motor task herein examined was non-fatiguing (i.e., 20%MVC to be maintained for approximately 15 to 20 s). Thus, we did not investigate aspects of motor control related to maintaining the torque target while preventing fatigue-related mechanical failure, which would intrinsically modulate the characteristics of motor units activation throughout the fatiguing contraction [61,62].

### 4.3. scTS for Neuromuscular Priming

Experimental and computational studies suggest that electrical stimulation of the spinal cord delivered with transcutaneous electrodes, or epidurally, activates common neural structures [63]. In particular, spinal stimulation recruits primarily large, myelinated fibres associated with somatosensory information at their entry into the spinal cord, which in turn engages the spinal circuitry controlling muscle activation [64]. Parameters of spinal stimulation play a key role in determining the characteristics of activation patterns facilitation, and we leveraged previous findings deriving from spinal cord injury research to define the spinal stimulation strategy implemented in this study [25]. Briefly, for each participant we initially determined the preferred cathode placement location between T11-T12 and T12-L1 spinous processes with the goal of maximizing the recruitment of both proximal and distal extensor motor pools. In fact, stimulation site has important implications for the topographical recruitment of neural structures associated with proximal and distal lower limb muscles [36]. Also, we selected 28 Hz as stimulation frequency considering our goal of increasing the excitability of lower limb extensor motor pools together with a larger neural network including interneurons. Previous research suggests that stimulation frequencies between 25 and 30 Hz can engage spinal networks involved in both tonic lower limb extension and rhythmic activation patterns, and that higher frequencies progressively promote the integration of interneurons [25]. Finally, stimulation intensity was always limited by participants’ comfort, and no visible lower limb muscle contraction was elicited by stimulation. This suggests that scTS neuromodulated the spinal circuitry without eliciting direct activation of motor pools.

In the present study, we explored the effects of scTS priming protocol on the characteristics of recruitment curves that can provide information on the excitability of the spinal circuitry (Figure 6). We observed statistical trends and medium effects of scTS priming protocol on lowering muscle activation threshold and increasing maximum amplitude of evoked potentials, which are both consistent with a trend of increased excitability of the spinal circuitry. These trends, together with the main findings of the study, are also in line with previous observations of the lasting effects of spinal stimulation (i.e., after its cessation) on the spinal circuitry controlling lower limbs [28,65,66]. It is also worth mentioning that the functional state of the spinal circuitry is dynamic and highly dependent on the afferent inputs [67]. On one hand, this suggests that the interpretation of the recruitment curve findings assessed in supine position (Figure 6) should be taken with a grain of salt when associated with the functional outcomes of the present study. On the other hand, this is the rationale that led us to apply scTS during an active neuromuscular priming protocol that provided weight bearing- and muscle contraction-related afferent inputs to the spinal cord, rather than at rest.

Future research efforts should be focused on implementing scTS with optimized exercise-based priming protocols that would not induce fatigue. This would allow us to investigate further if the proposed neuromodulation approach might support maximal lower limb motor output, and/or enhance training and rehabilitation programmes that include high-intensity neuromuscular efforts by counteracting central fatigue.

### 4.4. Limitations of the Study

A limit of the present study is that we did not assess the effects of sham priming protocol on the recruitment curve characteristics. Also, it is unclear whether an optimization of the exercise-based component of the priming protocol (i.e., which would not lead to decreased performance trends with sham stimulation) would have led to larger and significant performance improvements, rather than trends, when coupled with scTS. As mentioned above, another limit of this study is that we did not directly assess and quantify central and peripheral components of fatigue. Finally, it should be noted that the study participants were all young males who practiced sports that include aspects of lower limb explosive performance. At this stage, the findings herein reported may not be generalized to other populations, such as female and sedentary individuals.

## 5. Conclusions

In this study, we found significant interactions between scTS and sham priming protocols for relevant neuromuscular outcomes during maximal efforts of the lower limbs. The application of scTS during the proposed priming protocol tended to enhance or maintain relevant aspects of lower limb performance during maximal isometric and explosive efforts, which instead tended to impair after the same exercise-based protocol carried out with sham stimulation. Conversely, the control of a non-fatiguing submaximal motor task focused on steadiness of muscle torque generation was not affected by neither priming intervention. This suggests that scTS counteracted the unexpected fatigue induced by the exercise-based priming protocol by promoting positive, lasting adaptations in the nervous system controlling maximal efforts of the lower limbs in healthy active males.

## Figures and Tables

**Table 1:** Characteristics of the research subjects. BMI: body mass index. SD: standard deviation.

Subject	Age (Yrs)	Body Mass (Kg)	Stature (cm)	BMI (Kg/m^2^)	Sport Practiced	Training Activities (Days/Week)
1	23	74	169	25.9	Mixed martial arts	3
2	23	71	176	22.9	Resistance training	2
3	21	66	170	22.8	Resistance training	3
4	28	70	181	21.4	Fencing	2
5	23	96	184	28.4	Resistance training	2
6	22	73	175	23.8	Futsal	5
7	19	94	195	24.7	Basketball	2
8	22	73	174	24.1	Soccer	4
9	21	73	177	23.3	Soccer	4
10	24	91	182	27.5	Basketball	3
11	23	74	172	25.0	Soccer	5
12	23	75	181	22.9	Resistance training	4
Mean	22.7	77.5	178	24.4	-	3.3
SD	2.1	10.1	7.2	2.0	-	1.1

## Data Availability

The original contributions presented in this study are included in the article. Further inquiries can be directed to the corresponding author.
